# Supplementary material for: Arthrological reconstructions of the pterosaur neck and their implications for the cervical position at rest
Source: PeerJ. 2024 Feb 21;12:e16884. doi: 10.7717/peerj.16884 (PMC10893864; doi:10.7717/peerj.16884)
Supplement: Supplemental Information 1 — All measurements are in millimeters (mm) [file peerj-12-16884-s001.docx]

|  | Length (mm) | Height (mm) | Width (mm) |
| --- | --- | --- | --- |
| *Anhanguera piscator* |  |  |  |
| Atlas–axis | 35.0 | 50.9 | 38.6 |
| Cervical III | 61.7 | 51.1 | 47.8 |
| Cervical IV | 62.5 | 53.5 | 51.2 |
| Cervical V | 58.6 | 56.4 | 52.5 |
| Cervical VI | - | - | - |
| Cervical VII | - | 66.9 | - |
| Cervical VIII | 28.0 | 58.8 | 53.5 |
| Cervical IX | 26.4 | - | 73.6 |
| *Azhdarcho lancicollis* |  |  |  |
| Atlas–axis | 24.7 | 37.3 | 27.5 |
| Cervical III | 62.1 | - | 29.1 |
| Cervical IV | 83.6 | - | 36.0 |
| Cervical V | - | 18.7 | - |
| Cervical VI | 99.3 | 26.5 | 45.6 |
| Cervical VII | 50.0 | - | - |
| Cervical VIII | 43.0 | - | - |
| Cervical IX | - | - | - |
| *Rhamphorhynchus muensteri* |  |  |  |
| Atlas–axis | 7.6 | - | 6.1 |
| Cervical III | 7.8 | - | 7.1 |
| Cervical IV | 8.1 | - | 7.5 |
| Cervical V | 8.2 | - | 7.6 |
| Cervical VI | 7.9 | - | 7.5 |
| Cervical VII | 7.8 | - | 7.6 |
| Cervical VIII | 7.6 | - | 7.9 |
| Cervical IX | 6.9 | - | 8.5 |
|  |  |  |  |
